# Prevalence and Associated Factors of Odontogenic Maxillofacial Space Infections Among Adult Patients at Hawassa University Comprehensive Specialized Hospital, Ethiopia: A Retrospective Cross‐Sectional Study (2023–2025)

**DOI:** 10.1002/hsr2.72737

**Published:** 2026-06-30

**Authors:** Henok Tadesse, Fasika Kindae Yemer, Chala Ararsa, Wondimagegn Genaneh Shiferaw, Robel Mesfin, Bikila Lencha, Million Tadesse, Tagesse Taye Dayama, Mulugeta Edao Shate

**Affiliations:** ^1^ Department of Dental Medicine Hawassa University Hawassa Ethiopia; ^2^ Department of Emergency and Critical Care Nursing Wolaita Sodo University Sodo Ethiopia; ^3^ Department of Anesthesia and Anesthesiology, School of Medicine and Health Science University of Gonder Gonder Ethiopia; ^4^ Department of Epidemiology, School of Public Health, College of Health Sciences Madda Walabu University Shashemene Ethiopia; ^5^ Department of Anesthesia and Anesthesiology Hawassa University Hawassa Ethiopia; ^6^ Department of Nursing, School of Medicine and Health Science Hawassa University Hawassa Ethiopia

**Keywords:** associated factors, Ethiopia, maxillofacial space infection, odontogenic infections, prevalence

## Abstract

**Background:**

Odontogenic maxillofacial space infections are life‐threatening conditions that result from untreated dental problems, in which bacteria infect the facial fascial spaces from the affected teeth and supporting tissues. Despite their clinical severity, epidemiological data from sub‐Saharan Africa and Ethiopia remain limited. Understanding their prevalence and associated factors is essential for effective management and prevention.

**Objective:**

To determine the prevalence and associated factors of odontogenic maxillofacial space infection among adult patients (aged > 18 years) admitted to the Oral and Maxillofacial Surgery Unit at Hawassa University Comprehensive Specialized Hospital over 3 years (2023–2025).

**Methods:**

A retrospective cross‐sectional study using patients' medical records was conducted among patients admitted to the Oral and Maxillofacial Surgery between January 2023 and December 2025. Of these cases, 419 were deemed eligible according to predefined inclusion and exclusion criteria, and data were systematically extracted from the records. For a case to be included, there was a need to have clinical or radiographic evidence that the subject was infected and had pus drainage upon incision and drainage. Data were entered into Epi‐Data 4.6, and data analysis was carried out using SPSS Version 26.0 (Windows 10). Descriptive statistics, such as frequencies and percentages, and logistic regression analysis were done. *p* < 0.05 was considered statistically significant.

**Results:**

This study included 419 patient charts; 50 patients had confirmed odontogenic maxillofacial space infection. The prevalence of odontogenic maxillofacial space infection was 12.0% (95% CI: 9.0%–15.0%) among admitted patients. On multivariate analysis, rural residence (AOR = 2.70, 95% CI: 1.35–5.40), hypertension (AOR = 2.40; 95% CI: 1.15–5.00), diabetes mellitus (AOR = 3.10, 95% CI: 1.45–6.60), and delay in healthcare seeking (AOR = 4.00, 95% CI: 1.95–8.20) were independently associated.

**Conclusion:**

The prevalence of odontogenic maxillofacial space infections was relatively high among admitted patients in this tertiary hospital. Rural residence, hypertension, diabetes mellitus, and delay in healthcare seeking are associated with the infection. Strengthening early diagnosis and treatment of dental infections, improving access to oral health services, and encouraging timely healthcare‐seeking behaviour helps reduce the burden of odontogenic maxillofacial space infections.

**Abbreviations**
AOMIs: acute odontogenic maxillofacial infections
AOR: adjusted odds ratio
CI: confidence interval
COR: crude odds ratio
DNI: deep neck infection
HIV: human immunodeficiency virus
ICU: intensive care unit
IPD: inpatient department
LOH: length of hospitalization
ocmfs
: oral and craniomaxillofacial surgery
ODI: odontogenic infection
opd
: outpatient department
SIRS: Systemic Inflammatory Response Syndrome
WHO: World Health Organization

## 1 Introduction

Odontogenic infections are among the most prevalent bacterial infections affecting the maxillofacial region. These infections originate from dental tissues, such as dental caries, pulpitis, periodontal infection, or pericoronitis, and, in cases where treatment is insufficient, extend outside the alveolar bone into the adjacent fascial spaces, leading to odontogenic maxillofacial space infections [1, 2, 3]. Additionally, iatrogenic causes, such as complications following dental extractions or endodontic procedures, can also lead to these infections [4].

Odontogenic infection is the most common cause of deep neck infections in adults. Studies have also consistently demonstrated that mandibular molars are the most frequent source of odontogenic infections requiring hospital admission. In terms of fascial space involvement, the submandibular space is considered to be the most commonly affected site [5, 6]. Concerning the path of the disease, pus and bacteria travel from the subperiosteal or submucosal spaces to invade other spaces, such as the submandibular space. The submandibular space is one of the commonly involved deep fascial spaces, while the subperiosteal and submucosal spaces are early sites of the disease process [7].

Globally, odontogenic infections account for approximately 70%–95% of maxillofacial space infections, making them the leading cause of deep facial and neck infections [6, 8]. The anatomical spread of these infections is determined by the involved tooth, the position of the tooth apex in relation to muscle attachments, and facial plane pathways. International studies consistently report that mandibular teeth are responsible for about 60%–80% of odontogenic maxillofacial space infections, with mandibular molars, especially third molars, being the most common sources [9].

Factors contributing to this include poor oral hygiene practices, lack of awareness, substance use, limited availability of dental services, and cultural beliefs that delay healthcare‐seeking behaviors. In such regions, many patients present with advanced infections, which complicate management and increase the risk of adverse outcomes. These delayed presentations often result in prolonged hospitalization, increased healthcare costs, and a diminished quality of life for affected individuals [6, 8]. Odontogenic infections are usually treated by general dentists in the community. If it is untreated, it spreads beyond the confines of the bony maxilla or mandible, and it has the potential to result in life‐threatening complications [10, 11].

In Africa, odontogenic maxillofacial space infections remain a significant public health challenge, though high‐quality epidemiological data are limited. According to a study conducted in Ghana, the prevalence of odontogenic maxillofacial space infection was reported to be 5%–10% [12]. A similar study conducted in Nigeria reported 11.3% [13]. In developing countries, odontogenic infections remain a common cause of hospital admission due to limited access to dental care and delayed health‐seeking behavior.

However, evidence from Ethiopia remains limited and fragmented. Lack of local epidemiological data makes it difficult for healthcare providers and policymakers to design targeted prevention and treatment strategies. Therefore, this study aimed to determine the prevalence and associated factors of odontogenic maxillofacial space infection at Hawassa University Comprehensive Specialized Hospital, Hawassa, Sidama Region, Ethiopia.

## 2 Methods

### 2.1 Study Setting and Study Period

The study was carried out in the oral and maxillofacial surgery (OMFS) department, admitting inpatients at Hawassa University Comprehensive Specialized Hospital (HUCSH). HUCSH is located 275 km south of Addis Ababa and is 7 km southeast of the center of Hawassa city. It was established in 2006 and is one of the university hospitals in Sidama and the previous SNNPR. It has been serving more than 20 million people found in Sidama, SNNPR, Oromia, and some parts of the neighboring region. It provides high‐quality patient care in a broad range of general, specialty, and subspecialty levels of care, including maternal and child health services. Patient medical records from January 1, 2023, to December 31, 2025.

### 2.2 Study Design

An institution‐based retrospective cross‐sectional study was conducted.

### 2.3 Population

#### 2.3.1 Source Population

All patients admitted to the Oral and Maxillofacial Surgery Unit during the study period.

#### 2.3.2 Study Population

All selected adult patients (aged > 18 years admitted to the Oral and Maxillofacial Surgery Unit during the specified period at HUCSH and who met the eligibility criteria.

### 2.4 Eligibility Criteria

#### 2.4.1 Inclusion Criteria

People who were eighteen years old and above and went to the OMFS inpatient unit during the time we were studying were part of this study if their medical records had all the information we needed. We only looked at people who had an infection in the spaces of their face and jaw that started with a tooth problem. To be sure they really had this kind of infection we needed to see all of the following: when the doctor examined them they had symptoms like swelling, trouble opening their mouth pain and/or trouble breathing; x‐rays and/or computed tomography scans that showed something was wrong and when the doctor operated on them they found pus, which confirmed that the odontogenic maxillofacial space infection was really there.

#### 2.4.2 Exclusion Criteria

The following patients were not included in the study: Patients who were under 18 years old; patients with medical records or missing important information, where less than 80% of the required information was available. Patients with infections in the face and jaw area that did not come from problems, such as infections from the tonsils, trauma, salivary gland, or other non‐dental sources, and patients with infections caused by fungus. Patients who had surgery done at another hospital before coming to us and we could not get their medical records.

### 2.5 Sample Size and Sampling Procedure

#### 2.5.1 Sample Size

The sample size was determined by using a single population proportion formula. No previous study has been conducted in Ethiopia to determine the magnitude of odontogenic maxillofacial space infection among admitted patients.

$$n=\left({\text{Za}}^{2}\right)2\text{pq}/d^{2}$$

where *n* is the calculated sample size; *Z* = confidence interval [95%]; *p* = proportion [50%]; *d* = marginal error [5%]; *n *= (1.96)^2^ × 0.5 [1 − 0.5]/(0.05)^2^ *n* = 385.

Considering a 10% non‐response rate, the final sample size was 428.

#### 2.5.2 Sampling Technique and Procedure

The participants were chosen among patients who had been admitted to the Oral and Maxillofacial Surgery Unit in Hawassa University Comprehensive Specialized Hospital within the 3‐year study period. Based on the patient admission list at the hospital, there were 1286 patients who had been admitted to the hospital during this period. As the total number of patients admitted was larger than the required sample size, systematic sampling was used to obtain participants for the study. This involved choosing the sampling interval (*k*) by dividing the total number of patients admitted by the required sample size. Thus, every third record of patients admitted to the hospital was taken into consideration. Initially, one out of the first three records of patients was chosen randomly through the lottery method. Subsequently, every third record of patients was considered until a total of 428 patients' records were considered. The medical record numbers were found using the admission log book, and patients' records were examined accordingly. The first chart was selected randomly, then continued every third interval since *k* = 1286/428 = 3.

### 2.6 Data Collection Procedure and Tools

The data extraction checklist was adapted from previous studies conducted on odontogenic and maxillofacial space infections [3, 14]. A retrospective medical chart review was performed for all patients who were treated in the department during the selected time interval. Clinical charts and investigation reports were reviewed. The following variables were recorded systematically: socio‐demographic data (age, sex, and residence), clinical variables, anatomic variables (etiology, number and location of teeth involved, spaces involved, associated systemic diseases, previous antibiotic treatment), comorbidities, and other odontogenic maxillofacial space infections. Data were collected from patient medical records and hospital registration logs by trained data collectors under the supervision of the principal investigator.

### 2.7 Study Variables

#### 2.7.1 Dependent Variables

Odontogenic maxillofacial space infection (present/absent).

### 2.8 Independent Variable

Sociodemographic characteristics: Age, sex, and residence.

Anatomic site and involved tooth: Type of space, number of spaces, and type of tooth.

Medical factors: Diabetic mellitus, hypertension, and HIV/immunosuppression.

Clinical and Behavioral factors: Dental caries, smoking, alcohol, impaction, tooth trauma, delay in healthcare seeking, and previous dental treatment (RCT).

### 2.9 Case Definition and Variable Categorization

A “case” of OMSI was considered as an individual admitted into the OMFS inpatient ward who demonstrated clinically and radiologically evident infection of one or more fascial spaces of the maxillofacial region, with confirmation via incision and pus drainage. On the other hand, “non‐case” referred to any other OMFS admission, excluding the involvement of the fascial space. Age of individuals was classified into four categories, namely, 18–29, 30–44, 45–59, and 60+ years. Delayed access to medical care was determined when the patient had been admitted for 5 or more days since the beginning of the disease [15, 16].

### 2.10 Operational Definition of Terms

Maxillofacial space infection: Infection of the maxillofacial region due to odontogenic and nonodontogenic disease origins [8].

Odontogenic maxillofacial infections: Infection of the maxillofacial space due to dental disease [6, 8].

Delay in healthcare seeking: The time between the onset of dental infection symptoms and the patient's presentation to the hospital. Patients presenting more than 5 days after symptom onset were categorized as delayed healthcare seekers.

### 2.11 Data Quality Assurance

To ensure the quality of data collected from the study subjects, a range of mechanisms was employed to address major areas of bias. A 1‐day training was given for data collectors and supervisors on the objective and relevance of the study, how to gather the appropriate information, and the procedures of data collection techniques. The checklist was pre‐tested by taking 5% of the sample size in Adare General Hospital in Sidama Region. Necessary modifications in the checklist were made based on the nature of the gaps identified after the pretesting. The data collection process and completeness were closely supervised.

### 2.12 Data Analysis Procedure

The collected data were coded and entered into Epi‐Data 4.6, and Statistical data analysis was done using SPSS version 26.0 running on a Windows 10 operating system. Descriptive statistics like frequency and percentages were used to describe data. Categorical variables were expressed as percentages and continuous data as mean (±SD). The fitness of logistic regression models was assessed using the Hosmer–Lemeshow test, and its p‐value was 0.410. A Multicollinearity test was carried out to see the correlation between each independent variable, using the variance inflation factor (VIF) and a tolerance test, and there were no variables that did not meet the criteria. Variables with a *p*‐value < 0.25 in the bivariate analysis were entered into a multivariate logistic regression model to identify independent predictors. Odds ratios (ORs) with 95% confidence intervals (CIs) were calculated. Statistical significance was declared at *p* < 0.05.

### 2.13 Ethical Consideration

Ethical clearance was approved by the Institutional Review Board (IRB) of Hawassa University College of Medicine and Health Sciences with the reference number IRB/680/26 on 23 February 2026. A formal letter of permission was obtained from the HUCSH administration before data collection. Since the study was based on a retrospective review of patient charts, the requirement for informed consent was waived by the IRB. No personal identifiers (names and ID numbers) were collected; data were anonymized.

## 3 Results

### 3.1 Socio‐Demographic Characteristics of the Study Participants

A total of 428 patient records were targeted. Complete data were obtained for 419 patients, yielding a response rate of 97.9%. The mean age was 31.4 years (SD ± 12.7). The majority of participants were in the 30–44 age group. Males constituted 280 (66.8%) of the sample, and 245 (58.5%) lived in urban areas (Table 1).

**Table 1:** Socio‐demographic characteristics of respondents of patients admitted to the OMFS unit at a HUCSH in Sidama region (*N* = 419).

Variable	Category	Frequency	Percentage
Age	18–29	67	16.0
	30–44	209	49.9
	45–59	105	25.1
	≥ 60	38	9.0
Sex	Male	280	66.8
	Female	139	33.2
Residence	Urban	245	58.5
	Rural	174	41.5

### 3.2 Medical Factors of the Study Participants

About medical comorbidities among the 419 reviewed medical records. Diabetes mellitus was present in 24 patients (5.7%), while the majority (395, 94.3%) were nondiabetic. Hypertension was recorded in 40 patients (9.5%), and HIV/AIDS in 20 patients (4.8%) (Table 2).

**Table 2:** Medical factors among patients admitted to the OMFS Unit among attending HUCSH, Ethiopia (*N* = 419).

Variable	Category	Frequency (*n*)	Percentage (%)
Diabetes mellitus	Yes	24	5.7
	No	395	94.3
Hypertension	Yes	40	9.5
	No	379	90.5
HIV/AIDS	Yes	20	4.8
	No	399	95.2

### 3.3 Clinical and Behavioral Factors of the Study Participants

About the clinical conditions among the 419 study participants. Dental caries in 73 patients (17.4%). Impacted tooth was present in 150 patients (35.8%), previous dental treatment (including root canal therapy) in 80 patients (19.1%), and tooth trauma in 50 patients (11.9%) (Table 3).

**Table 3:** Predisposing and Behavioral factors among patients admitted to the OMFS Unit among attending HUCSH, Ethiopia (*N* = 419).

Variable	Category	Frequency (*n*)	Percentage (%)
Dental caries	Yes	73	17.4
	No	346	82.6
Impacted tooth	Yes	150	35.8
	No	269	64.2
Previous dental treatment	Yes	80	19.1
	No	339	80.9
Tooth trauma	Yes	50	11.9
	No	369	88.1
Delay in healthcare seeking	> 5 days	121	28.9
	< 5 days	298	71.1
Tobacco smoking	Yes	63	15.0
	No	354	85.0
Alcohol use	Yes	105	25.1
	No	314	74.9

### 3.4 Anatomic Factors Among Infected Patients

Among the 50 patients who had odontogenic infection in their maxillofacial space, the submandibular space was found to be the most frequently affected anatomical space, with 29 patients (58%) affected. The second most common space affected is the buccal space, involving 15 patients (30%). The sublingual space and submental space affect 10 patients (20%) and 8 patients (16%), respectively. The least frequently involved deep space infection is the parapharyngeal space, infecting 4 patients (8%).

As regards involvement of more than one anatomical space, the majority (65.4%) of the cases had infections restricted to one anatomical space only, while 9 patients (34.6%) had infections involving two or more spaces.

In relation to the teeth serving as the source of infection, mandibular molars are the most common origin, with 35 cases (70%). The mandibular premolars follow, with 8 patients (16%) being affected. Five patients (10%) have maxillary molars as the source of infection, while the least common source is maxillary anterior teeth with 2 patients (4%) affected (Table 4).

**Table 4:** Anatomic factors among infected patients (*n* = 50).

Anatomic factor	Category	Frequency (*n*)	Percentage (%)
Space involved (multiple may apply)	Submandibular	29	58.0
	Buccal	15	30.0
	Sublingual	10	20.0
	Submental	8	16.0
	Parapharyngeal	4	8.0
Number of spaces	Single	33	66.0
	Multiple (≥ 2)	17	34.0
Tooth source	Mandibular molar	35	70.0
	Mandibular premolar	8	16.0
	Maxillary molar	5	10.0
	Maxillary anterior	2	4.0

### 3.5 Prevalence of Odontogenic Maxillofacial Space Infection Among OMFS Admissions

**Figure 1:**
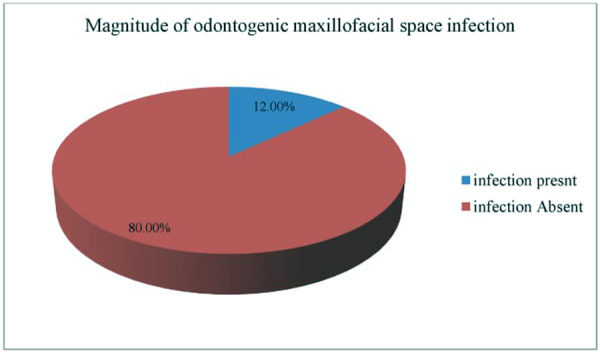
Magnitude of odontogenic maxillofacial space infection.

Of the 419 OMFS admissions reviewed, 50 patients had confirmed odontogenic maxillofacial space infection, giving an overall magnitude of 12% (95% CI: 9.0%–15.0%). The figure specifically reflects the proportion of OMFS inpatient admissions with confirmed OMSI and should not be interpreted as representing the general hospital population (Figure 1).

### 3.6 Factors Associated With the Odontogenic Maxillofacial Space Infection

In bivariable logistic regression analysis, seven variables were found to be associated with odontogenic maxillofacial space infection at *p* < 0.25, including: residence, diabetes, sex, delay in healthcare seeking, dental caries, and hypertension. After controlling for potential confounders, four variables—residence, delay in healthcare seeking, Hypertension, and diabetes—were significantly associated with odontogenic maxillofacial space infection in multivariable logistic regression at 95% CI with a *p*‐value < 0.05.

Patients living in rural areas had 2.7 times higher odds of developing odontogenic maxillofacial space infection compared to urban residents (AOR = 2.70; 95% CI: 1.35–5.40). Similarly, diabetic patients had three times higher odds of infection compared to non‐diabetic patients (AOR = 3.10; 95% CI: 1.45–6.60). Hypertensive patients had 2.4 times higher odds of infection compared to normotensive patients (AOR = 2.40; 95% CI: 1.15–5.00).

In addition, patients who waited more than 5 days before seeking hospital care had four times higher odds of infection compared to those who presented within 5 days (AOR = 4.0; 95% CI: 1.95–8.20) (Table 5).

**Table 5:** Bivariate and multivariate analysis of factors associated with infection.

Variable	Category	Yes (*n* = 50)	No (*n* = 369)	COR (95% CI)	AOR (95% CI)	*p* value
Residence	Rural	31	143	2.38 (1.05–5.40)	2.70 (1.35–5.50)	0.005*
	Urban	19	226	1	1	
Diabetes	Yes	15	9	10.61 (4.03–27.9)	3.10 (1.45–6.60)	0.003*
	No	35	360	1	1	
Hypertension	Yes	12	28	3.17 (1.20–8.37)	2.40 (1.15–5.00)	0.019*
	No	38	341	1	1	
Sex	Male	38	242	1.66 (0.84–3.29)	1.50 (0.50–3.10)	0.28
	Female	12	127	1	1	
Delay in healthcare seeking	Yes	35	86	6.36 (2.68–15.1)	4.00 (1.95–8.20)	< 0.001*
	No	15	283	1	1	
Dental caries	Yes	19	54	3.58 (1.89–6.79)	1.30 (0.60–2.60)	0.49
	No	31	315	1	1	

Abbreviations: AOR, adjusted odds ratio; CI, confidence interval; COR, crude odds ratio.

* Statistically significant at p < 0.05.

## 4 Discussion

The current research determined the prevalence and associated factors for odontogenic maxillofacial space infection in hospitalized individuals at the Oral and Maxillofacial Surgery Unit of Hawassa University Comprehensive Specialized Hospital. This study found that 12% of admitted patients had odontogenic maxillofacial space infection, and rural residence, diabetes mellitus, hypertension, and delayed health care seeking behaviour were significantly associated with odontogenic maxillofacial space infection.

The prevalence of odontogenic maxillofacial space infection was 12%. This is consistent with the Nigerian (11.3%) and Indian (12.8%) studies [13, 17]. It is slightly higher than the 5%–10% reported from Ghana [12]. This might reflect differences in healthcare access, referral patterns, or study populations. The inclusion of only admitted patients in this study may also influence the observed magnitude, as it primarily reflects more severe cases requiring hospitalization.

Of the five fascial spaces involved, the submandibular space (58%) was the most commonly involved, reflecting the evidence indicating that the roots of mandibular molars, especially those affected by periapical pathologies, have an anatomical location such that they drain to the submandibular space following infection progression from the subperiosteal and submucosal spaces. Mandibular molars were the most frequent odontogenic tooth type causing OMSI (70%), reflecting the greater literature, which shows that mandibular molars affect up to 60%–80% of cases [9]. Out of the 50 OMSI cases, dental caries were identified in 19 (38%) as being the primary etiological cause. This is less common than the incidence of caries as an etiological factor in the existing literature on the subject, with caries reported as the most common etiology in OMSI cases. However, this is likely due to the fact that dental caries were not the documented causative agent in the patient files but rather the disease state resulting from dental caries.

In this study, rural residence was associated with odontogenic maxillofacial space infection. Patients living in rural areas were nearly three times more likely to develop an infection compared with urban residents. This finding is similar to a study conducted in India [18]. This might be limited access to dental care, lower oral health literacy, longer travel distances, and cultural beliefs that delay seeking care. Rural patients need targeted outreach: mobile dental clinics, training of health extension workers, and subsidized transportation to tertiary centers.

Another important factor identified in this study was diabetes mellitus. Diabetic patients had three times higher odds of infection compared to non‐diabetic patients. This study is similar to a study conducted in China [19]. This might be hyperglycemia, which impairs immune function and tissue healing, allowing infections to progress rapidly. Screening diabetic patients for early odontogenic infections should be a priority. From a clinical perspective, this observation stresses the need for checking whether a patient has diabetes before treating any case of infection of the facial spaces, in addition to managing his/her blood sugar level optimally.

The present study found that patients who presented to the hospital more than 5 days after symptom onset were four times more likely to develop odontogenic maxillofacial space infection compared to those who presented within 5 days. This finding is consistent with previous studies conducted in India and Nigeria [15, 16]. This could be the unavailability of proper dental care, ignorance regarding the signs of infection, and financial issues, especially among patients living in rural areas. In addition, some patients might resort to self‐treatment at the initial stages of the infection, thus delaying their visit to a doctor. These results demonstrate the need to enhance community awareness regarding early dental care, along with providing better access to dental care services, particularly in rural areas. Health practitioners must focus on the early treatment of dental infections and refer the patient to a specialist as soon as possible.

Hypertension was also identified as a factor associated with odontogenic maxillofacial space infection in this study. This was consistent with the study conducted in India [7]. This could be due to vascular compromise, similar risk factors, or the presence of more comorbidities among those suffering from hypertension. Clinically, healthcare providers should carefully evaluate patients with systemic comorbidities and provide early management of dental infections to reduce the risk of complications.

The findings of this study are significant in terms of clinical practice and public health considerations. In terms of clinical practice, early screening of patients suffering from systemic diseases for dental pathology, as well as the referral process in case of suspicion of fascial space infection, can help prevent further disease development. From the perspective of policy makers, the high correlation with rural living as well as late presentation is indicative of the need for provision of primary dental care and health education in such populations.

## 5 Strengths and Limitations of the Study

### 5.1 Strengths

This study has several important strengths. First, the study provides valuable information on the prevalence and associated factors of odontogenic maxillofacial space infections in a tertiary hospital setting in Southern Ethiopia, where limited evidence is currently available. The findings contribute to the existing body of knowledge and may help guide clinical practice and public health interventions aimed at preventing severe odontogenic infections. Second, the study included a relatively adequate sample size and applied appropriate statistical methods, including both bivariable and multivariable logistic regression analyses, to identify independent factors associated with the infection. This analytical approach helped to control potential confounding variables and improve the reliability of the findings.

Third, the study used hospital medical records covering a defined study period, which allowed for the assessment of real clinical cases managed in a specialized referral hospital. This enhances the clinical relevance of the findings for healthcare providers managing patients with odontogenic infections.

### 5.2 Limitations

First, the study used a retrospective cross‐sectional design based on medical record review, which may be subject to incomplete or missing data due to documentation limitations in patient records.

Second, since the study was conducted in a single tertiary hospital, the findings may not be fully generalizable to other healthcare settings, particularly primary healthcare facilities or other regions of Ethiopia.

Third, some potentially important variables, such as oral hygiene practices, socioeconomic status, nutritional status, and health‐seeking behavior, were not consistently documented in the medical records and therefore could not be included in the analysis. The absence of these variables may limit the ability to fully explain all factors associated with odontogenic infections.

Finally, because of the cross‐sectional nature of the study, it was not possible to establish a causal relationship between the identified factors and the occurrence of odontogenic maxillofacial space infections.

## 6 Conclusion and Recommendations

### 6.1 Conclusion

The prevalence of odontogenic maxillofacial space infection among adult patients admitted to the oral and maxillofacial surgery unit at HUCSH was 12%. Rural residence, hypertension, diabetes mellitus, and delayed healthcare seeking (> 5 days from symptom onset) were independently associated with odontogenic space infection.

### 6.2 Recommendations

Based on the findings, the following actions are recommended.

#### 6.2.1 For the Sidama Regional Health Bureau and HUCSH Administration

Establish and strengthen primary dental clinics in rural districts to enable early treatment of caries and pericoronitis. Integrate routine oral health screening into hypertension and diabetes care programs. Develop a public awareness campaign using local media (radio, community meetings), emphasizing that facial swelling requires care within 5 days.

#### 6.2.2 For Healthcare Providers at HUCSH

To screen all patients presenting with facial space infections for diabetes and record the duration of symptoms. Provide discharge counseling on oral hygiene and early signs of recurrence.

#### 6.2.3 For Future Research

To conduct prospective and multi‐center studies to further investigate the clinical outcomes and determinants of prolonged hospital stay among patients with odontogenic maxillofacial space infections.

## Author Contributions

**Henok Tadesse:** conceptualization, methodology, software, data curation, investigation, formal analysis, supervision, writing – original draft, writing – review and editing. **Fasika Kindae Yemer:** investigation, methodology, software, supervision, visualization. **Chala Ararsa:** conceptualization, methodology, software, data curation, formal analysis, supervision, writing – review and editing. **Wondimagegn Genaneh Shiferaw:** conceptualization, investigation, methodology, validation, software, data curation, supervision. **Robel Mesfin:** investigation, methodology, validation, visualization, software, supervision. **Bikila Lencha:** conceptualization, methodology, software, data curation, writing – review and editing, visualization, investigation, supervision. **Million Tadesse:** conceptualization, software, methodology, formal analysis, visualization, writing – review and editing, investigation, writing – original draft. **Tagesse Taye Dayama:** conceptualization, software, formal analysis, supervision, writing – review and editing, visualization, data curation, methodology. **Mulugeta Edao Shate:** conceptualization, methodology, software, data curation, formal analysis, supervision, visualization, writing – original draft, writing – review and editing, investigation.

## Funding

The authors have nothing to report.

## Ethics Statement

This study was conducted in accordance with the Declaration of Helsinki. Ethical approval was granted by the Hawassa University College of Medicine and Health Sciences (Ref. No: IRB/680/26). Informed consent was waived due to the retrospective use of anonymized medical records.

## Conflicts of Interest

The authors declare no conflicts of interest.

## Transparency Statement

The corresponding author, Henok Tadesse, affirms that this manuscript is an honest, accurate, and transparent account of the study being reported; that no important aspects of the study have been omitted; and that any discrepancies from the study as planned have been explained.

## Data Availability

We described all the relevant information in the manuscript, but the refined dataset can be obtained from the corresponding author upon reasonable request.
